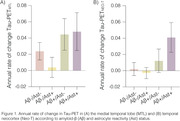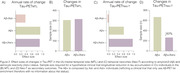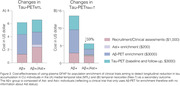# Plasma GFAP use for population enrichment of clinical trials in preclinical Alzheimer’s disease

**DOI:** 10.1002/alz.092554

**Published:** 2025-01-09

**Authors:** Bruna Bellaver, Guilherme Povala, Pamela C.L. Ferreira, Guilherme Bauer‐Negrini, Firoza Z Lussier, Douglas Teixeira Leffa, João Pedro Ferrari‐Souza, Matheus Scarpatto Rodrigues, Carolina Soares, Markley Silva Oliveira, Nesrine Rahmouni, Cécile Tissot, Joseph Therriault, Stijn Servaes, Jenna Stevenson, Andrea L. Benedet, Nicholas J. Ashton, Rebecca E. Langhough, Tobey J. Betthauser, Bradley T. Christian, Rachael E Wilson, Henrik Zetterberg, Kaj Blennow, Dana Tudorascu, Eduardo R. Zimmer, Thomas K Karikari, Pedro Rosa‐Neto, Sterling C. Johnson, Tharick Ali Pascoal

**Affiliations:** ^1^ University of Pittsburgh, Pittsburgh, PA USA; ^2^ Department of Psychiatry, University of Pittsburgh School of Medicine, Pittsburgh, PA USA; ^3^ Universidade Federal do Rio Grande do Sul, Porto Alegre, Rio Grande do Sul Brazil; ^4^ Federal University of Rio Grande do Sul, Porto Alegre, RS Brazil; ^5^ Translational Neuroimaging Laboratory, The McGill University Research Centre for Studies in Aging, Montreal, QC Canada; ^6^ Lawrence Berkeley National Laboratory, Berkeley, CA USA; ^7^ Translational Neuroimaging Laboratory, The McGill University Research Centre for Studies in Aging, Montréal, QC Canada; ^8^ Department of Psychiatry and Neurochemistry, Institute of Neuroscience and Physiology, The Sahlgrenska Academy, University of Gothenburg, Mölndal, Gothenburg Sweden; ^9^ Department of Psychiatry and Neurochemistry, Institute of Neuroscience and Physiology, The Sahlgrenska Academy, University of Gothenburg, Mölndal Sweden; ^10^ Wisconsin Alzheimer’s Institute, University of Wisconsin‐Madison School of Medicine and Public Health, Madison, WI USA; ^11^ School of Medicine and Public Health, University of Wisconsin‐Madison, Madison, WI USA; ^12^ Department of Medical Physics, University of Wisconsin‐Madison School of Medicine and Public Health, Madison, WI USA; ^13^ Wisconsin Alzheimer's Institute, University of Wisconsin School of Medicine and Public Health, Madison, WI USA; ^14^ Department of Psychiatry and Neurochemistry, Institute of Neuroscience and Physiology, The Sahlgrenska Academy at the University of Gothenburg, Gothenburg Sweden; ^15^ Institute of Neuroscience and Physiology, The Sahlgrenska Academy at the University of Gothenburg, Mölndal Sweden; ^16^ Federal University of Rio Grande do Sul (UFRGS), Porto Alegre, RS Brazil; ^17^ University of Pittsburgh School of Medicine, Pittsburgh, PA USA

## Abstract

**Background:**

We showed that plasma GFAP (a proxy of astrocyte reactivity) abnormality is key to unleashing Aβ effects on tau phosphorylation in preclinical AD. This suggests that selecting cognitively unimpaired(CU) individuals with both high Aβ and plasma GFAP could offer an early time window in the disease, but with an increased risk of developing tau pathology. Here, we tested the utility of plasma GFAP for population enrichment in clinical trials focusing on CU individuals.

**Method:**

We assessed 195 CU individuals from TRIAD (n=84) and WRAP (n=111) cohorts with plasma GFAP and Aβ‐PET at baseline and Tau‐PET ([^18^F]MK6240) at baseline and follow‐up. The annual rate of progression in Tau‐PET was measured as follow‐up minus baseline uptakes divided by time between scans. Aβ positivity was determined as Centiloid>20. Astrocyte reactivity positivity(Ast+) was defined based on plasma GFAP of younger Aβ‐. Effect size was calculated as the mean change in Tau‐PET divided by the SD. Estimated sample size was calculated testing a hypothesized 25%drug effect on tau accumulation reduction in the medial temporal lobe (MTL) and temporal neocortex (Neo‐T) as secondary outcomes with 80% power at a 0.05 level.

**Results:**

A significant annual rate of MTL tau‐PET accumulation was observed in both Aβ+/Ast‐ and Aβ+/Ast+ groups (Figure 1A), while Neo‐T Tau‐accumulated only in the Aβ+/Ast+ group (Figure 1B). The Aβ+/Ast+ group has a larger effect size on Neo‐T tau accumulation (0.97) compared to Aβ+/Ast‐ (0.33) and all individuals Aβ+ (0.61; Figure 2C). Plasma GFAP as an enrichment strategy reduced the sample size of clinical trials using changes in the Neo‐T as outcome in 60% (Figure 2D), with 59% reduction in clinical trial costs (Figure 3B). Plasma GFAP as an enrichment strategy for changes in MTL tau accumulation did not provide significant advantages over using Aβ only (Figure 2‐3). Larger effect sizes in Tau‐PET accumulation were observed in WRAP than TRIAD cohort.

**Conclusion:**

The use of Aβ+/Ast+ for population enrichment would reduce up to 59% clinical trial costs aiming to detect changes in Tau‐PET compared to using Aβ only. To conclude, clinical trials focusing on preclinical AD would benefit from enrolling individuals with both Aβ pathology and astrocyte reactivity to select individuals enhancing trial cost‐effectiveness.